# Reframing neglect in neglected tropical diseases and related environmental determinants: A scoping review of photovoice studies

**DOI:** 10.1371/journal.pntd.0014655

**Published:** 2026-08-21

**Authors:** Noor Adilla Md Anuar Hussain, Mohd Rohaizat Hassan, Azmawati Mohammed Nawi, Aliff Faisal Ahmad Kamar, Shahrul Azhar Md Haniff, Fateen Nadhira Ismail

**Affiliations:** 1 Department of Public Health Medicine, Universiti Kebangsaan Malaysia, Kuala Lumpur, Malaysia; 2 Centre for Research Development, University of Cyberjaya, Selangor, Malaysia; 3 Borneo Medical and Health Research Centre, Faculty of Medicine and Health Sciences, University Malaysia Sabah, Kota Kinabalu, Sabah, Malaysia; Kwame Nkrumah University of Science and Technology, GHANA

## Abstract

**Background:**

Neglected tropical diseases (NTDs) affect over one billion people worldwide and are closely linked to environmental determinants, including unsafe water, poor sanitation, inadequate housing, and vector-borne exposure. Yet, the voices of affected communities remain insufficiently centred in research or policy. Photovoice, a participatory visual method, has potential to reframe the neglect of NTDs by centring affected communities and their lived experiences. This scoping review aims to explore the application of photovoice in NTD research and its related environmental determinants.

**Methodology/findings:**

Six electronic databases were searched using terms related to photovoice and NTDs and reported in accordance with the PRISMA Extension for Scoping Reviews. Data from thirteen included studies were extracted and synthesised using thematic analysis. Most studies were conducted in Africa and focussed on skin-related NTDs. Photovoice methods were adopted across studies, including variations in participant training, equipment, and discussion formats, while retaining its core principles of ethical practice, participant-driven image production, and reflective dialogue. The degree of participation and power-sharing varied across studies. Community members were engaged as co-researchers to differing extent, with uneven inclusion across gender and age groups and limited transparency in shared analysis and decision-making. Reported outcomes included increased visibility of lived experiences, strengthened participant confidence, enhanced community dialogue, locally initiated actions, and contributions to programme reflection or policy discussions. Nevertheless, few studies implemented dissemination strategies such as exhibitions, community meetings, booklets, videos and policy dialogues to create visibility, which limits opportunities to influence policy.

**Conclusion/significance:**

Photovoice in NTDs research and related environmental and vector-borne determinants demonstrates the potential to reframe neglect by successfully transforming invisibility into evidence and turning community voices into advocacy, while inclusive participation, skilled facilitation, and structured pathways are needed in future studies to translate this visibility into lasting policy impact.

## Author summary

Neglected tropical diseases (NTDs) affect millions of people, primarily in marginalised communities, yet the experiences of those affected are often overlooked in research and policy. Photovoice is a participatory research method that allows community members to use photographs to document and share their daily health challenges. This scoping review of 13 studies from Africa and Asia found that photovoice increased visibility of lived experiences, strengthened participant confidence, and encouraged dialogue and locally led actions. However, only a few studies implemented dissemination strategies such as exhibitions, community meetings, booklets, videos, or policy dialogues, limiting opportunities to influence policy. In conclusion, photovoice can reframe neglect in the context of NTDs and related environmental determinants that shape exposure to NTDs, but future research must prioritise inclusive participation, skilled facilitation, and clear pathways to translate findings into lasting policy impact.

## Introduction

Neglected tropical diseases (NTDs), comprising 20 diseases recognised by the World Health Organization (WHO), continue to affect over one billion people globally [1]. The burden is greatest among low-income, marginalised, and rural communities in which illness is compounded by systemic neglect, stigma, and inequities [2]. These affected communities often live in environments where access to safe water, sanitation, and hygiene (WASH) is inadequate, and they also encounter recurrent exposure to disease vectors, and close human–animal interaction that increase zoonotic risks [3]. Together, these environmental and vector-borne determinants create interconnected conditions that sustain the transmission and persistence of NTDs.

Neglect in the context of NTDs is a product of the structural invisibility of affected populations [4]. NTDs thrive in settings of poverty, fragile health systems, and the lack of political influence, leaving them in the shadow of high-profile diseases such as HIV, tuberculosis, and malaria [2]. Inadequate investment in research and development, poor policy prioritisation, and the stigma attached to those people affected reinforce their marginalisation. This cycle of neglect keeps the lived realities of affected people marked by disability, poverty, and exclusion, remaining largely absent from policy debates and global health agendas. Overcoming neglect therefore requires approaches that expose these realities and amplify the voices of those affected, rather than relying solely on biomedical interventions [5].

Traditional research approaches rarely reflect the lived realities of affected populations, who were rarely engaged in the creation of knowledge [6,7]. As a result, policy responses risk perpetuating top-down interventions that ignore the structural conditions that perpetuate disease. Community-based participatory research (CBPR) offers an alternative by shifting power towards communities and valuing local knowledge in public health decision-making [8]. Photovoice, a participatory visual methodology introduced by Wang & Burris, exemplifies this shift [9]. By equipping affected communities with cameras and developing platforms for critical reflection, photovoice enables them to document, interpret, and communicate their lived experiences [10,11]. Beyond its role in data collection, photovoice is a participatory tool that acts as a platform for reflection, dialogue, advocacy, and engagement with decision-makers [12,13].

As photovoice gains traction within public health, there is an opportunity to examine how this participatory approach has been applied in NTDs research and related environmental and vector-borne determinants, and how it may help reframe neglect and promote equity in global health. Given the emerging and methodologically diverse nature of photovoice research in this field, a scoping review approach was used to map the available evidence. Specifically, this scoping review aimed to describe the contexts in which photovoice has been used, with particular attention to the populations involved, methodological adaptations, participatory depth, reported outcomes, and implications for future public health research, practice and policy. The review was guided by three questions:

1. What NTDs and related environmental or vector-borne determinants have been examined using photovoice methods?
2. How has photovoice been operationalised in these studies, including participant groups, training, data generation, facilitation, analysis, and dissemination?
3. What forms of community insight, public health relevance, policy linkage and reported impact have emerged from photovoice studies in this field?

## Methodology

### Ethics statement

This scoping review used data from previously published literature and did not involve the direct recruitment of human participants. Therefore, institutional ethical approval was not required for this review. Ethical approval and informed consent procedures were reported variably across the included studies by the respective original authors. Studies involving adult participants generally described informed consent procedures, while studies involving children additionally reported parental or legal guardians’ consent and child assent, where applicable, in accordance with the requirements of the approving institutional review boards or ethics committees.

This review was conducted as a scoping review using systematic search, screening, data charting, and synthesis methods. Reporting was guided by the Preferred Reporting Items for Systematic Reviews and Meta-Analyses Extension for Scoping Reviews (PRISMA-ScR). The review protocol was registered in PROSPERO (CRD420251039130). A scoping review approach was considered appropriate because the purpose was to map the extent, characteristics, and applications of photovoice in research on NTDs and related environmental or vector-borne determinants, rather than to estimate intervention effectiveness or generate pooled effect estimates.

### Preferred reporting items for systematic reviews and meta-analyses extension for scoping review (PRISMA-ScR)

PRISMA-ScR was introduced in 2018 as a reporting guideline specifically designed for scoping reviews [14]. It provides two main advantages which include 1) enhancement of research transparency and credibility by requiring authors to report 20 key items, and 2) provision of structured guidance tailored specifically to scoping reviews rather than depending on PRISMA, the systematic review standards [15]. In this review, the PRISMA-ScR framework enabled a rigorous and reproducible search process to identify photovoice applications in NTDs and their associated environmental determinants.

### Search strategy and information sources

The literature search was conducted in March 2025 using six electronic databases to obtain adequate coverage of the peer-reviewed literature: Web of Science (WoS), Scopus, PubMed, Ovid, SAGE Journals, and EBSCOhost. The search strategy was developed through review of relevant literature, thesaurus, and expert consultation. Search terms were organised into three core concepts: (1) neglected tropical diseases and related environmental or vector-borne determinants, (2) photovoice or participatory visual methods, and (3) public health action, participation, or reported outcomes. Boolean operators and database-specific syntax were adapted for each database. Full database-specific search strategies are provided in Supporting Information (S1), with a simplified PubMed search string presented in Table 1.

**Table 1:** Simplified PubMed search string.

Database	Keyword Used
PubMed	(“Neglected Tropical Disease*” OR NTD* OR “Chagas Disease” OR “Human African Trypanosomiasis” OR Leishmaniasis OR “Taenia solium cysticercosis” OR Taeniosis OR Dracunculiasis OR Echinococcus OR “Foodborne trematod*” OR “Lymphatic Filariasis” OR Onchocerciasis OR Schistosomiasis OR “Soil-Transmitted Helminth*” OR Ascariasis OR “Hookworm Disease*” OR Trichuriasis OR Strongyloidiasis OR “Buruli Ulcer” OR Leprosy OR Hansen OR Trachoma OR Yaws OR Bejel OR Pinta OR Dengue OR Chikungunya OR Rabies OR Mycetoma OR Chromoblastomycosis OR “Deep Mycosis” OR Scabies OR Myiasis OR “Snakebite Envenoming” OR “environmental determinant*” OR “vector-borne determinant*” OR WASH OR sanitation OR housing OR “water exposure” OR malaria)AND(Photovoice OR “photo voice” OR “participatory photography” OR “visual participatory method*” OR “community photography” OR “action research with photography” OR “visual advocacy”)AND(Prevent* OR Control OR Change OR Empower* OR “community engagement” OR “community participation” OR “community-based intervention*” OR advocacy OR “health promotion” OR “policy influence” OR “programme influence”)

### Eligibility criteria

Studies were eligible if they were original research articles that applied photovoice or closely related participatory visual methods to NTDs or related environmental and vector-borne determinants. No restrictions were imposed on the date of publication or language. Although malaria is not classified as an NTD, malaria-related studies were considered eligible only if they examined related environmental or vector-borne determinants relevant to community-based disease prevention, health equity, or disease control in neglected settings. The full inclusion and exclusion criteria are summarised in Table 2

**Table 2:** The inclusion and exclusion criteria.

Criterion	Eligibility	Exclusion
Literature type	Original research	Reviews, books, book chapter, editorial letters
Methodological approach	Studies using photovoice or similar participatory visual methods	Studies using researcher-generated images without participatory interpretation
Disease focus or Environmental determinants	• Studies on WHO-recognised NTDs, or • Studies addressing environmental determinants that shape exposure to NTDs, including: ○ Water, Sanitation, and Hygiene (WASH) ○ Vector-borne disease risk (mosquitoes and flies) ○ Zoonotic disease risk	Studies not relevant to NTDs or their associated environmental or vector-related transmission pathways

### Study selection

All retrieved records were exported into Microsoft Excel, and duplicates were removed by checking titles, authors, years, and digital object identifiers where available. Screening was performed in two stages. First, titles and abstracts were independently screened by two reviewers (NA and AF) using predefined eligibility criteria. Second, the full texts of potentially eligible articles were retrieved and independently assessed by the same two reviewers. Discrepancies between reviewers during both screening phases were resolved through discussion and consensus with arbitration by a third reviewer (MRH) where necessary.

### Data extraction

Data from included studies were extracted using a structured data charting form developed for this review. The variables extracted in accordance with the review questions included: (i) author(s), publication year and country; (ii) NTD focus or associated environmental determinant; (iii) participant characteristics; (iv) description of photovoice purposes and methodology applied, and (v) reported outcomes. Data charting was conducted by NA and independently verified by MRH for accuracy, consistency, and completeness. Any discrepancies were resolved through discussion and consensus. The charting form was refined during the process to ensure that relevant methodological and outcome-relevant information was captured consistently across included studies.

### Data synthesis

An inductive thematic approach was used to synthesise findings. The synthesis focussed on how photovoice was applied, the populations and disease or determinant contexts involved, methodological adaptations, participatory depth, reported outcomes, and implications for public health practice and policy. Patterns were compared across studies to identify common applications, methodological variations, forms of community insight, and reported links to programme or policy influence. Given the heterogeneity of study aims, designs, and outcomes, no meta-analysis was undertaken.

### Quality appraisal

Methodological quality was appraised using the Mixed Methods Appraisal Tool (MMAT). This tool measures study quality across research designs such as qualitative, quantitative, and mixed methods studies. Nevertheless, the appraisal was not used to exclude studies, consistent with the mapping purpose of this scoping review. Instead, the MMAT findings were used to support interpretation of the credibility, transferability, and reporting strength of the included studies. This process identified that all 13 articles are of high-quality, with no studies excluded based on appraisal outcomes, as shown in Table 3.

**Table 3:** Quality appraisal result based on MMAT.

Qualitative study									
Author (Year)	Are there clear research questions?	Do the collected data allow to address the research questions?	Is the qualitative approach appropriate to answer the research question?	Are the qualitative data collection methods adequate to address the research question?	Are the findings adequately derived from the data?	Is the interpretation of results sufficiently substantiated by data?	Is there coherence between qualitative data sources, collection, analysis and interpretation?	Criteria met	Overall quality
Koffi et al. (2018) [13]	✓	✓	✓	✓	✓	✓	✓	5	High
Mtuy et al. (2021) [16]	✓	✓	✓	✓	✓	✓	✓	5	High
Adekeye et al. (2023) [17]	✓	✓	✓	✓	✓	✓	✓	5	High
Chowdhury et al. (2023) [18]	✓	✓	✓	✓	✓	✓	✓	5	High
Dien et al. (2023) [19]	✓	✓	✓	✓	✓	✓	✓	5	High
Levison et al. (2012) [20]	✓	✓	✓	✓	✓	✓	✓	5	High
Bisung et al. (2015) [21]	✓	✓	✓	✓	✓	✓	✓	5	High
Bisung et al. (2015) [22]	✓	✓	✓	✓	✓	✓	✓	5	High
Barakat & Caprara (2021) [23]	✓	✓	✓	✓	✓	✓	✓	5	High
Naserrudin, Lin, et al. (2023) [24]	✓	✓	✓	✓	✓	✓	✓	5	High
Naserrudin, Yong, et al. (2023) [25]	✓	✓	✓	✓	✓	✓	✓	5	High
**Mixed method**									
**Author_Year**	**Are there clear research questions?**	**Do the collected data allow to address the research questions?**	**Is there an adequate rationale for using a mixed methods design to address the research question?**	**Are the different components of the study effectively integrated to answer the research question?**	**Are the outputs of the integration of qualitative and quantitative components adequately interpreted?**	**Are divergences and inconsistencies between quantitative and qualitative results adequately addressed?**	**Do the different components of the study adhere to the quality criteria of each tradition of the methods involved?**	**Criteria met**	**Overall quality**
Krentel et al. (2024) [26]	✓	✓	✓	✓	✓	✓	✓	5	High
Onyango et al. (2025) [27]	✓	✓	✓	✓	✓	✓	✓	5	High

## Results

**Fig 1:**
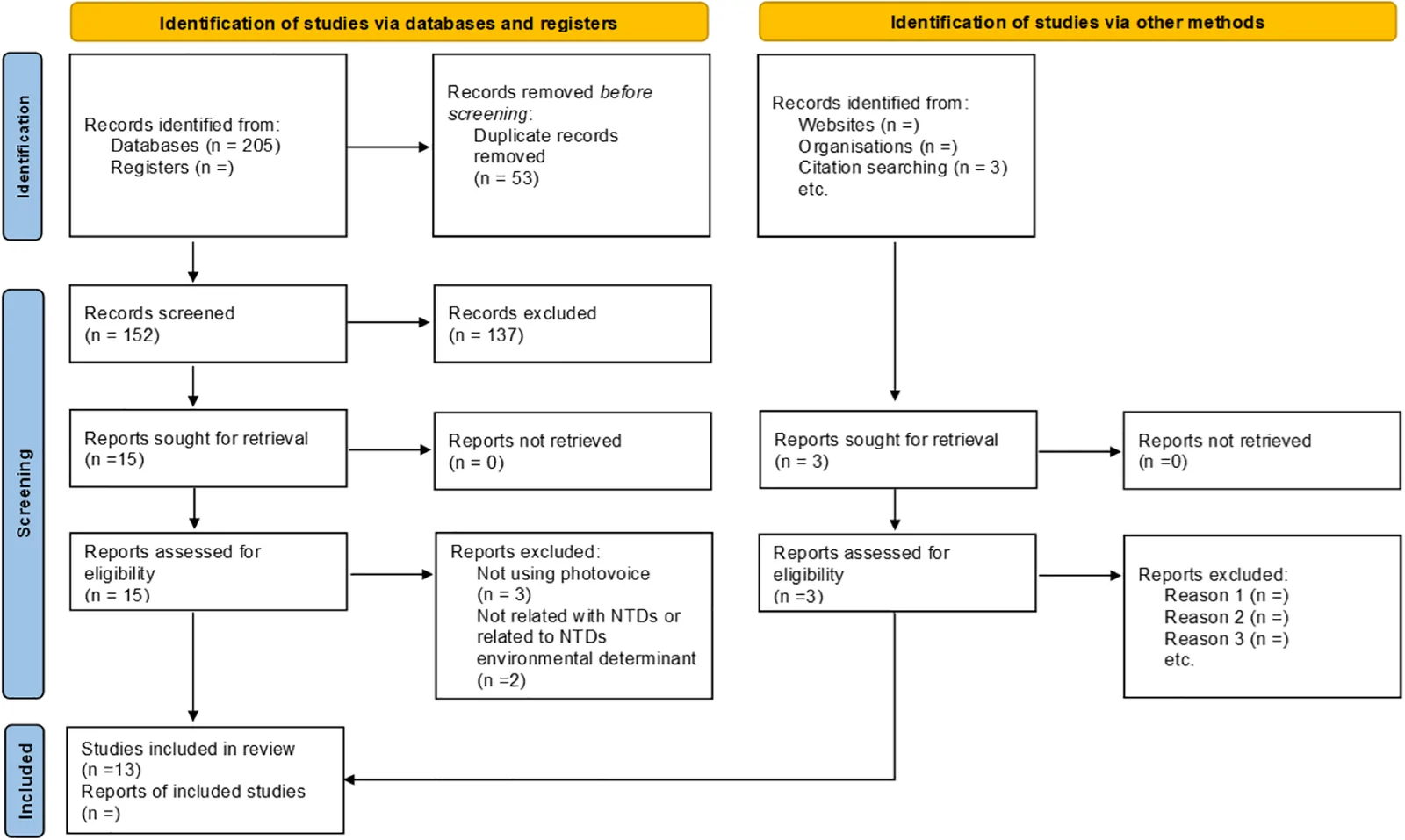
PRISMA flow diagram of study selection process.

The initial total of 208 records was found, of which 205 were from database searches and an additional three records from citation searches. After removing duplicates (*n* = 53), 155 records remained for title and abstract screening, of which 137 were excluded for failing to meet the inclusion criteria. In total, 18 full-text articles were assessed in detail. Of these, five were excluded for not using photovoice (*n* = 3) or not addressing NTDs or their environmental determinants (*n* = 2). Ultimately, 13 studies met the eligibility criteria and were included in the review. The complete results of the screening and selection process are presented in Fig 1.

### Characteristics of studies included

Thirteen studies were included in this review. The studies were published between 2012 and 2025, with a surge in publications after 2020, reflecting an increased interest in photovoice as a participatory approach in NTD-related public health research. Most studies were carried out in Sub-Saharan Africa (*n* = 9), including Kenya (*n* = 4), Nigeria (*n* = 2), Tanzania (*n* = 1), Côte d’Ivoire/Mauritania (*n* = 1), and Ghana–Madagascar (*n* = 1). Three studies were conducted in Southeast Asia, including Malaysia (*n* = 2) and Indonesia (*n* = 1). One study was conducted in Latin America (Brazil). Six studies used photovoice to directly examine NTDs, including skin NTDs, schistosomiasis (including female genital schistosomiasis (FGS)) and trachoma [13,16–19,26]. The remaining seven studies examined related environmental or vector-borne determinants, including WASH practices (*n* = 3) [20–22], and vector-borne determinants (*n* = 4) [23–25,27]. Although malaria is not classified as an NTD, these studies were retained because they addressed shared environmental and vector-borne determinants relevant to disease prevention and control in neglected settings. Methodologically, most studies used qualitative community-based participatory designs (*n* = 12), including two studies that combined photovoice with survey or mixed-methods components [18,20]. A detailed summary of the study characteristics, photovoice purposes, participants, implementation approaches and major reported insights is presented in Table 4.

**Table 4:** Summary characteristics of included studies in systematic review.

Author (Year), Country	NTD focus	Photovoice purpose	Participants	Methodology of photovoice implementation	Major insights/ outcomes
Photovoice studies addressing Neglected Tropical Diseases (NTDs)					
Koffi et al. (2018), Côte d’Ivoire and Mauritania [13]	Schistosomiasis	To assess knowledge, attitudes, and practices (KAP) of communities towards schistosomiasis in two rural settings.	*n* = 32; community members from two rural sites; gender not specified.	Mixed-methods; KAP household questionnaires and photovoice. Selected individuals from household were given disposable cameras to take any images related to schistosomiasis. Participants briefed on ethics and asked to remain anonymous; no duration or cycles reported. Field assistants received 2 days of training. Participants were interviewed individually and in group utilising SHOWeD prompts and conducted in local language to explore meanings and messages. Photos were then analysed to determine knowledge and strategies to control the disease in the community	Findings showed that most participants had heard of schistosomiasis and identified symptoms such as blood in urine or stool, abdominal pain, and fatigue. Local terms framed the disease, with causes attributed to dirty water, sun, animal urine, witchcraft, and mysticism, besides misconceptions such as sexual transmission and contaminated food persisted. Reported prevention practices included avoiding rivers and lakes, using wells or taps, boiling water, handwashing, and not urinating/defaecating in water, though the practices were inconsistently implemented. Communities depended heavily on self-treatment and traditional medicine, as the use of formal healthcare was limited by cost, distance, and drug shortages. The photovoice technique revealed evident gaps due to a disconnect between biomedical knowledge and culturally rooted beliefs, as well as highlighting the need for context-specific health education.
Mtuy et al. (2021), Tanzania [16]	Trachoma	To document Maasai women’s lived experiences of educating the community after trachoma control training	*n* = 20; all Maasai women from three villages	CBPR photovoice with Maasai women. A combined trachoma-control and camera with ethics workshop was the kick-off for the activity. Participants were asked to distribute messages from the workshops in their communities, and to record their experiences using disposable cameras. As requested by the participants, household-head permission was obtained prior to camera use. A week-1 follow-up to assure support. The capture window was 2 weeks; cameras were then returned to develop the film. At week 5, participants were given the prints, chose 2 images (one “success,” and one “challenge”), and participated in group dialogues using SHOWeD in order to reflect and suggest actions. Findings and photos were showcased in public exhibitions aimed at local leaders.	Photovoice empowered women and made them into educators and change agents. Community and leader engagement followed, with visible actions such as new handwashing stations at homes and schools. Women provided practical inputs for trachoma-control messaging, illustrating high knowledge retention and contextual insight, in which they spontaneously identified animal carcases as fly-breeding sites beyond information given in the workshops. Participants were proud and developed confidence in using cameras; they were identified as health advocates. At first, people mistrusted cameras, and the women responded by explaining and involving ethical practice to alleviate public concern and build trust.
Adekeye et al. (2023), Nigeria [17]	Skin NTDs	To describe the daily challenges and coping of affected people and their caregivers.	*n* = 21; 14 affected; 7 caregivers; mixed gender	Qualitative photovoice with a 1-day briefing on ethical photography, camera use, and core themes (stigma, well-being, and health). Participants (people affected by skin NTDs and caregivers) photographed their daily challenges and coping mechanisms. Using a five-step process, they combined individual and group elicitation sessions (SHOWeD-style) to interpret images, followed by co-selecting key photos with validated captions. Co-researchers facilitated analysis and collated outputs into photo booklets for dissemination.	Participants experienced compounded stress due to the poverty, stigma, and disability. Livelihoods were precarious, including selling livestock, stopping work/school, and occasional begging. Enacted stigma (shunning, ridicule, discrimination) and internalised stigma (concealment; feeling useless, shame, sadness) were pervasive, especially with visible morbidity, driving depression, anxiety, low self-esteem, and isolation. Gendered harms included fear of being abandoned and the inability to fulfil provider/caregiver roles. Despite this, coping strategies, such as faith, peer and family support, safe spaces, vocational training and microcredit restored dignity, strengthened self-efficacy and enhanced mental well-being. The findings led to an impetus for support groups, as well as referral linkages, which strengthened the psychosocial care along with clinical services.
Chowdhury et al. (2023), Nigeria [18]	Skin NTDs	To document support group leader experiences’ of facilitating support groups and their impact on peer-led engagement and community inclusion	*n* = 10; support group leaders affected by skin NTDs; gender and age not specified	CBPR with photovoice embedded. A 1-day group training was conducted to introduce photovoice, camera use and research ethics. Participants were provided with photo prompts about well-being, stigma, services, and support-groups impact. They then captured some images in their communities. Next, individual elicitation interviews were conducted to explore meanings. Participants presented some of the photos, refined narratives, and finalised captions in the group sessions. Key images were categorised into sub-themes (stigma, well-being, support/services). Findings from dialogues were triangulated with programme data. Results were fed back to communities, and a photo booklet was put together for dissemination.	Photovoice captured increased self-efficacy and peer solidarity, with leaders describing visible changes among members. Evidence showed decreased stigma, restored confidence, economic empowerment, and enhanced case detection through support-group networks. Insights led to practical programme adjustments and paved the way for stigma reduction activities. Sustained by local collaboration and income-generating activities, the groups have become self-owned platforms for healing, advocacy, and long-term community impact.
Dien et al. (2023), Indonesia [19]	Leprosy	To increase public and policy awareness and alleviate stigma.	*n* = 30; mixed gender, persons affected with leprosy, their family members and healthcare workers	CBPR photovoice using ethical training and stigma-sensitive protocols. Participants were guided to photograph daily activities, health routines and living conditions to capture concerns, struggles, and strengths through iterative image capture. They then joined facilitated group reflections to add narratives and captions and to select three to five important photos for each. A public World Leprosy Day exhibition attracted over 250 people (community leaders, NGOs, and government officials). Images were also compiled into a citywide video to help extend outreach and impact.	Photovoice helped people living with leprosy, their families and healthcare workers to reveal hidden struggles and resilience. Participants felt heard and validated, had improved confidence and reduced internalised stigma, and increased social participation. A public exhibition developed community empathy and brought understanding to leaders and policymakers. The visual narratives catalysed local advocacy and demonstrated the value of photovoice as a tool for health promotion and engagement in policy processes that amplify the voice of marginalised communities to create social change.
Krentel et al. (2024), Ghana and Madagascar [26]	Female genital schistosomiasis (FGS)	To explore emotional and social experiences of women of reproductive age with FGS and inform integrated FGS services.	Number of participants not reported; Women of reproductive age in districts with more than 50% prevalence of schistosomiasis	Mixed-methods Photovoice with women of reproductive age during endline assessment. After an orientation, which included ethical guidance, participants used objects and scenes in their immediate surroundings to symbolically represent their perceptions and experiences of schistosomiasis and FGS. Photo-capture was coupled with group reflection dialogues, in which images were shared and interpreted. Selected images and insights were then used in endline dissemination to stakeholders to inform FGS integration within services.	Women employed visual metaphors to reveal gendered stigma and emotional burden (dried leaves for shame and ill-health; abandoned buildings for social exclusion). The process fostered participatory learning, community dialogue, and awareness-building around women’s health and inclusion. Participants took on advocacy roles and raised the visibility of FGS. Insights supported integration of FGS within primary care platforms and generated programmatic learning to inform policy uptake.
**Photovoice on Environmental and Vector-Borne Drivers related to NTDs**					
Levison et al. (2012), Kenya [20]	Environmental determinant (WASH)	To identify problems in daily behaviours related to the water and sanitation that affect the health of the community for the development of community-led solutions.	*n* = 25; 8 men, 17 women aged more than 18 years old; village residents.	The mixed methods involved community mapping and photovoice. A 1-day basic photography training (disposable cameras) preceded fieldwork. Participants then photographed for 1 week: water sources; purification/treatment; storage, collection, and transfer; sanitation facilities; plus, context features (employment sites, housing, animals, family/community, and firewood collection). Total: 582 photos. Images were discussed in group dialogues, followed by stakeholder debriefs. Photo analysis was performed on the listed categories and stratified by gender and age (younger versus older than 40 years old).	Photovoice revealed behaviour-health relationships frequently overlooked by interviews and defined priorities for action. Images were clustered around people (26.8%), activities around water (26.6%) and homes (20.9%). Gender patterns were distinct - women focussed on domestic tasks and lake-water collection; while men focussed on income activities (e.g., fishing). Age patterns also differed; younger participants were more concerned with children (20.6%) and shoreline water use, while older participants recorded wells (4%) and adults (14%). Photos showed routine high-risk co-location of bathing, laundry, and drinking water collection at the same contaminated lakeshore, indicating how gender, age, and livelihood influence daily water-health risk. These visual insights enabled concrete prioritisation and paved the ways for action on safer collection points, segregation implementation and targeted behaviour change messaging.
Bisung, et al. (2015); Kenya [21]	Environmental determinant (WASH)	To examine how socio-political, environmental and gendered factors influence water-health linkages.	*n* = 8 All were women, aged between 22 and 54.	The mixed methods involved community mapping and photovoice. Training was provided in DhoLuo on ethics and camera use. Participants were given disposable cameras and went on photo-walks to record water and sanitation practices that impact health. Over 8 days, each captured 16–26 images and selected 4 for the interview session. Thirty-two interviews (60–90 minutes) explored meaning, relevance, and practical solutions to each image. A group discussion brought together the individual insights and aided action planning. Findings were reported back to the community in a meeting/exhibit to generate collective decisions and next steps.	Photovoice identified unsafe WASH behaviours and structural limitations. Images recorded open-defaecation areas, poorly constructed pit latrines, industrial pollution of Lake Victoria, and sand harvesting that created mosquito-breeding sites. The burden fell heaviest on women and children (pregnant women with ~30 L; children missing school to go fetch water). Households reported high water costs (~USD 1.25 per person/day) in the face of unemployment. Community responses, e.g., CHV-led hygiene education and women’s-group mobilisation, initiated local WASH actions but were circumscribed by unequal resources, weak government support, and low trust in leadership. Overall, photovoice enhanced community voice with authorities, facilitating tangible advocacy and at the same time exposing embedded vulnerabilities visually.
Bisung et al. (2015), Kenya [22]	Environmental determinant (WASH)	To explore KAP and empower women in a lakeshore community.	*n* = 8; all women aged 28–55; included 2 community health volunteers.	Qualitative photovoice. Training and ethics were given in the local language. After consent, were informed of research ethics, camera usage, and photo-taking. Over 8 days, each captured 16–26 images on WASH practices affecting health. They then joined individual interviews and group discussions (English and DhoLuo) in order to interpret the images; each choosing four photos for more in-depth dialogue. Analysis informed a community feedback meeting with provincial leaders, health officials, schoolchildren, and community leaders to share findings, gather community input, and promote community-led WASH solutions. Soap bars were distributed as a hygiene reminder as well as a sign of appreciation.	Photovoice revealed structural constraints to safe water and sanitation and the extent of community agency, and provided evidence for targeted WASH advocacy and infrastructure asks. Three outcomes ensued: (1) awareness of ignored hazards (open defaecation, unsafe co-use of contaminated water); (2) spontaneous action in the field (informal community education); (3) planned interventions (engage elderly, improve latrines, mobilise households).
Barakat & Caprara (2021), Brazil [23]	Vector borne (Dengue)	To increase awareness and build knowledge for community surveillance in the school environment and understand student viewpoints on vector control.	*n* = 55; school students (Grades 4–9); gender not specified.	School health–promotion project with photovoice elements. Following workshops on vector control and photovoice, students (with the authorisation of the school board) were given an orientation on ethics and image capture. With mobile phones or digital cameras, they took pictures of risk settings in and around the school. Four focus groups were set up to discuss images; the sessions were recorded on tape, which were transcribed and thematically analysed using content analysis by Minayo. Strict ethics were emphasised including informed consent/assent of students and guardians, privacy safeguards, and voluntary participation. The findings were shared with the school and community for local surveillance and action.	Photovoice enhanced vector habitat recognition by uncovering overlooked breeding grounds and instigating critical thinking and an expression of responsibility among students. The process increased awareness of environmental issues, related health and education, and mobilised school-community surveillance. Students became agents of local surveillance and sustaining health promotion.
Naserrudin, Lin, et al. (2023), Malaysia [2 4]	Vector borne – (Zoonotic Malaria)	To explore barriers and facilitators to *Plasmodium knowlesi* malaria prevention.	*n* = 26 (4 male, 22 female, aged 21–68 years old); rural villagers	CBPR photovoice after stakeholder engagement and ethics approval. Villagers were recruited through introductory workshops, received ethical training, and provided informed consent. Participants held three rounds of photography and FGDs, each 20–30 days apart. Sessions used SHOWeD prompts to reflect on malaria causes, prevention challenges, and community recommendations. FGDs were held in Sabah Malay, with Rungus translation where necessary. Questions were piloted locally for clarity. Participants chose their own photos for discussion. There was member-checking in the final round. Participants were given phone credits. Photo-sharing was done with the participants’ consent (some anonymous, some named). Dissemination included a community photo exhibition with health officers and local leaders, villagers, and academics, as well as a World Malaria Day 2022 showcase with the Ministry of Health and policymakers.	Through 215 participant-generated photographs and 3 rounds of focus group discussions, this photovoice study found that despite the widespread knowledge that *P. knowlesi* malaria is transmitted through mosquito bites from macaques, major barriers exist to protective behaviours. Low perceived threat, dissatisfaction with preventive tools, and questions about the efficacy of tools restricted consistent use. Daily livelihoods like farming, fishing, hunting and cultural practices such as social gatherings often took place outdoors at night, where exposure is increased. Structural challenges such as poor housing, unreliable water supply, lack of electricity, and mosquito breeding sites further compounded risks. Facilitators included a high level of malaria awareness, fear of hospitalisation, social support networks, gendered household roles which kept women indoors and trusted healthcare services for bed nets and regular education.
Naserrudin, Yong, et al. (2023), Malaysia [25]	Vector borne – (Zoonotic Malaria)	Document local knowledge on malaria causation and prevention practices of rural communities	*n* = 26 adults (4 men and 22 women, aged 21–72 years);4 rural villages	The CBPR photovoice approach involved five phases. First, the identification of sites and participants. Second, ethic orientational training was presented in the Sabah Malay language (with translation of Rungus available). Consent processes included identifiable images; there was no guidance from the photographer to maintain participant perspectives. Third, a 14-day photo-capture period using participants’ own smartphones. Participants documented behaviours, environments, and metaphoric representations of malaria exposure and mosquito avoidance. Fourth, three FGDs per village (12 in total) used SHOWeD prompts to discuss malaria knowledge, preventive practices and challenges. Discussions were held in Sabah Malay, with Rungus translation. Participants selected images and provided narratives. Finally, selected images were printed and securely archived; key photos were disseminated in exhibitions with stakeholders, including policymakers.	Across three iterations of photography-FGD cycles, participants demonstrated clear causal models, i.e., malaria is transmitted through mosquito bites carrying *Plasmodium* from monkeys (zoonotic *P. knowlesi*). They mapped multi-level risks -- environmental (rainy season, stagnant water, deforestation, and monkey intrusion), behavioural (outdoor livelihoods and low repellent/bed net use), and socioeconomic (poverty, poor waste disposal, and poor local health services) factors. Modern repellents were considered expensive or ineffective; traditional deterrents (garlic, lemongrass and smoke from the coconut husks) and protective clothing were widely used. New knowledge emerged around mosquito-repelling plants. Communities implicated monkey incursions in habitat disruption, and urged humane management. The photovoice process built local voice, allowed structured dialogue with policymakers via public exhibition and facilitated the integration of community perspectives into malaria control.
Onyango et al. (2025), Kenya [27]	Vector borne – (ivermectin mass drug administration [MDA] for malaria control)	Understand participants’ experiences in the BOHEMIA clinical trial (ivermectin MDA trial) and their perception of the effects of Ivermectin MDA on individuals and the community to inform acceptability and implementation.	10 adults (6 men, 4 women); clinical trial participants.	Photovoice was nested in the social-science arm of the BOHEMIA RCT across five Kenyan villages at/after MDA round 3. Participants completed a 1-day training (ethics, camera use, and research purpose) and provided consent. Subsequently, they used digital cameras for 2 weeks to photograph responses to two prompts: (1) trial experiences and (2) perceived effects of the MDA. They subsequently participated in two picture-sharing group discussions that were separated by trial arm (intervention vs. control), each presenting five photos per prompt and explaining their selections. Discussions took place in Kiswahili, audio-recorded with consent, transcribed, anonymized and analysed; findings were fed back in community feedback sessions.	Participants expressed high levels of confidence in trial processes due to the following visible safeguards: weight-based dosing, household barcode tagging, informed consent, pregnancy testing, and directly observed therapy. The photos provided evidence of perceived benefits, i.e., increased appetite, fewer mosquitoes, clearing of bedbugs and jiggers, improvement of ringworm infection, and minor negative effects (rashes and dizziness). Implementation challenges arose: hard-to-reach households, flooding-related displacement, and a lack of male support for pregnancy testing for women. Participants questioned sustainability without addressing environmental sources (mosquito breeding sites and contaminated water). Overall, photovoice captured lived realities, enabled participants to bring to light feasibilities and gender concerns and provided tangible evidence to improve acceptability messaging and MDA roll-out.

**Fig 2:**
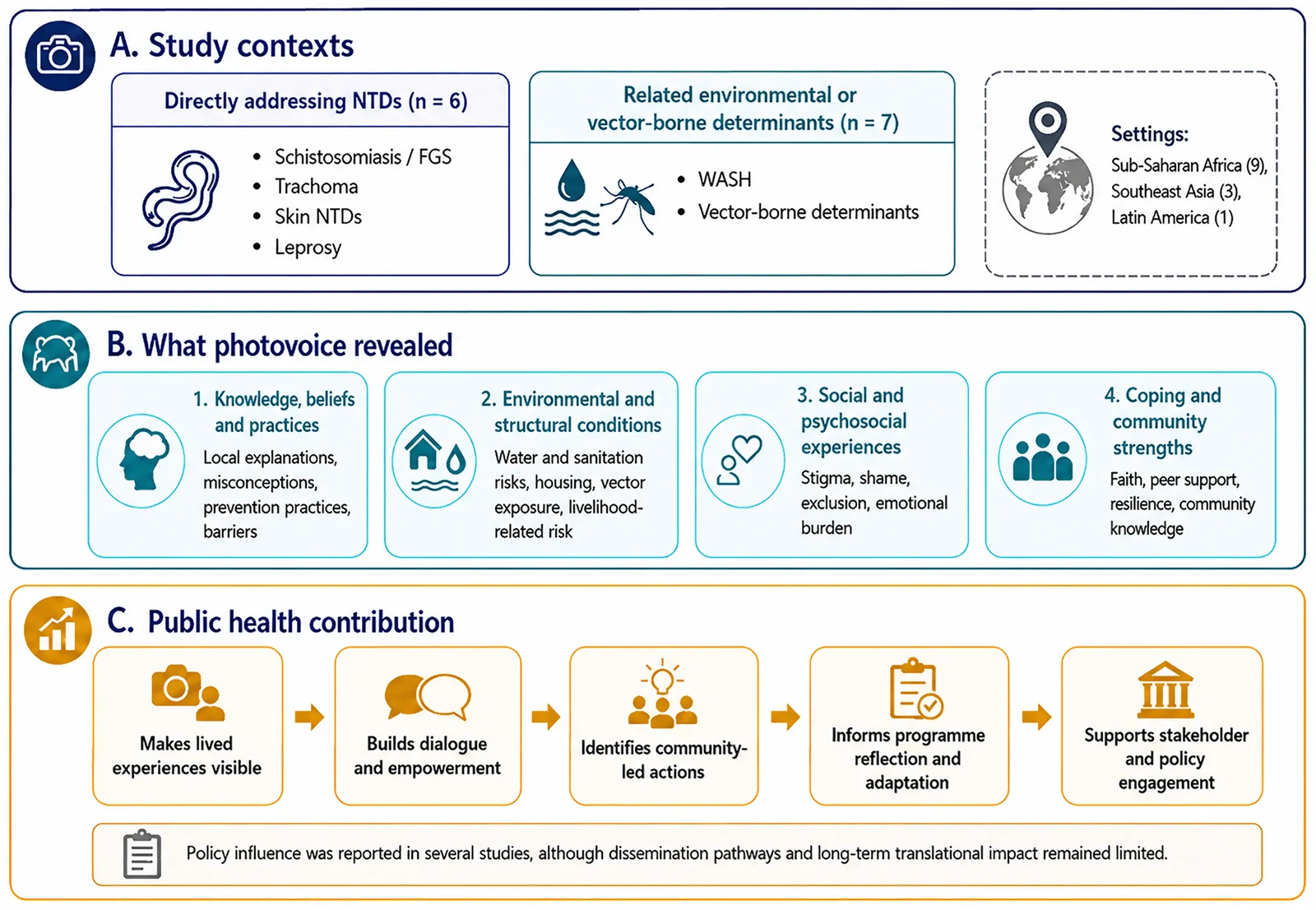
Photovoice applications in research on NTDs and related environmental or vector-borne determinants.

Building on the detailed study characteristics presented in Table 4, the key thematic pathways through which photovoice has contributed to research on NTDs and their related environmental determinants are mapped in Fig 2.

### Adaptation to the photovoice method

Photovoice was adopted with flexibility but retained its key principles of community photography, group reflection, and dialogue. All studies were initiated with training sessions covering camera use, basic photography, and ethics, including consent for images that could be identified. The training duration ranged from single-day workshops to multi-week trainings. The equipment used differed; six studies offered disposable or instant cameras, three used digital cameras, and three used smartphones. Meanwhile two studies used mixed approaches, such as WhatsApp for sharing photos and continuous conversation [25,27]. The photo-taking periods lasted from less than a day to several weeks. One study had three (3) iterative cycles of photo-taking and group discussion [24]. Participants tended to choose up to 10 photographs for analysis. Thirteen studies (93%) used the SHOWeD framework to guide interpretation and meaning-making. Reflection was mediated through individual interviews, group discussions, or a combination of both. Analytical strategies included thematic analysis, content analysis, and photo analysis. Most used transcripts as their primary data, but several used photographs as their core analytical material. Dissemination went beyond journal articles: nine studies hosted exhibitions or community events, three developed photo-booklets, and one created video documentation to bring results back to participants and stakeholders.

### Participation of participants in photovoice processes

Most studies recruited adults aged 18 years and above. Only one study in Brazil involved younger participants, i.e., elementary schoolchildren from Grades 4–9 [23]. Nine studies included both men and women, while three studies were undertaken on women only [16,21,26]. Sex distribution was not reported in two studies. Sample sizes ranged from 8 to 56 participants, and the most common sampling strategy was purposive sampling. Participant composition varied, including persons affected by NTDs, caregivers, community health volunteers, villagers, schoolchildren, and frontline health workers. Eleven studies (79%) explicitly targeted marginalised groups such as women, rural villagers, and people living with stigmatising skin conditions. The purpose of involvement also differed between studies. Five studies used photovoice to identify problems and lived realities, such as stigma and barriers to prevention [17,19,24,25]. Two studies explored knowledge, attitudes, and practices (KAP) around NTDs and their determinants [13,22]. Another three used it as a health promotion or empowerment tool [16,26,27], and two evaluated the acceptability and effects of the intervention [26,27].

### Outcome of the photovoice process

Photovoice research produced results at the individual, community, and policy levels, as presented in Table 5.

**Table 5:** Thematic outcomes of the photovoice process.

Outcome	Theme	Findings
Individual level	Empowerment and increased confidence	In Ghana and Madagascar, the women in the FGS and Trachoma project took on advocacy roles, challenging gender norms and raising awareness in the community [16,27].
	Improved awareness and knowledge	People affected by leprosy, Buruli ulcer, and other skin NTDs reported a lower level of internalised stigma, increased social participation, and improved understanding of NTD causes, prevention, and health-seeking practices [13,17,19,22]
Community level	Community dialogue and discussion	Stimulated conversation on WASH and environmental risks such as sanitation, water use, malaria prevention, and distrust of health systems [13,22,26]
	Collective action	Community-led initiatives such as the formation of support groups in Nigeria and WASH initiatives in Kenya [6,20]
Programme / Policy level	Advocacy and engagement	Findings translated into exhibitions, booklets, or policy briefs that engaged health authorities, NGOs, and local leaders. In Tanzania, photo exhibitions helped empower Maasai women as trachoma advocates [16]
	Programme evaluation	Visual narratives informed the acceptability, communication, trust and implementation challenges related to the trial of ivermectin MDA in Kenya [27]

## Discussion

This scoping review focuses on photovoice application in NTDs research and their related environmental and vector-borne determinants, with particular attention to its capacity to reframe neglect by making marginalised experiences visible and actionable. Guided by the framework of Wang & Burris, previous studies were evaluated to determine whether they achieved the three foundational aims of photovoice: (1) recording and reflecting community realities, (2) promoting critical dialogue, and (3) reaching policymakers [11]. When these aims are achieved, an issue and a population that was once invisible becomes visible, reframing neglect and positioning NTDs as issues that cannot be ignored.

### Recording and reflecting community realities

Photovoice has long been applied across health, education, and social justice to bring hidden realities to the surface and promote the agency of participants [28–31]. Consistent with evidence from broader public health and social research, photovoice in NTD-related studies enabled participants to document environmental, social, and behavioural factors that sustain disease transmission. Visual narratives captured key environmental risks, including stagnant water, unsafe sanitation, housing conditions, and daily practices linked to malaria, zoonotic exposure, and WASH-related transmission, while also revealing psychosocial dimensions, such as stigma, caregiving burdens, and gendered responsibilities [20,25]. The process of taking photos made participants more aware of risky behaviours they previously had not noticed or been normalised to, such as open defaecation and lack of water treatment [21]. In a study on trachoma, Maasai women were documented as they made efforts to educate others, recording their successes promoting hygiene with clean faces, compound cleaning, and leaky-tin handwashing stations and their struggles as community health ambassadors [16]. The process helps participants to think critically and develop a deeper understanding of the problems and possible solutions for their community [32]. Through such narratives, photovoice achieved the first aim of *documenting community strength and concern.*

Yet, important gaps remain. Although skin NTDs formed one important subset of the included studies, photovoice was also applied to schistosomiasis, FGS, trachoma, dengue-related determinants, WASH-related determinants, and malaria-related environmental or vector-borne determinants. This suggests a relatively diverse but still small evidence base. Less visible but equally burdensome NTDs remain under-represented, particularly where disease experience and transmission pathways are not easily captured through external visual documentation [33].

Participation was also uneven across gender and age groups. The participant selection varied according to study objectives, disease focus, and photovoice design. Some studies intentionally recruited specific groups, such as women, young people, people affected by skin NTDs or communities exposed to environmental risks, while others included broader community samples. These patterns suggest that, without deliberate attention to inclusivity, photovoice may reproduce existing silences rather than challenge them, limiting its ability to fully reframe neglect. Without attention to diversity, photovoice runs the risk of becoming tokenism that is valued for its participatory label but does not shift power or create meaningful change [34]. Future applications must therefore extend disease focus, diversify participation, and embed photovoice in iterative cycles that capture evolving risks and responses. Only then can photovoice go beyond documenting to transformation and sharpen its role as a tool for confronting inequities and disrupting the structural neglect that sustains NTDs.

### Promoting critical dialogue through group discussion of photographs

Critical dialogue involves engaging with different perspectives to encourage mutual understanding and critique. It is not simply about exchanging views but about probing and critically examining the values and assumptions that underpin the views [35]. Group discussion of photographs was central to translating individual observations into shared understanding [36]. In Kenya, images of unsafe lake washing stimulated women to organise a *baraza* (community meeting) with the elders and chiefs, translating recognition into concrete plans for provision of well covers, latrine closures, and community clean-ups [21]. In Tanzania, Maasai women organised village discussion groups to share trachoma photographs with mothers and grandmothers, transforming educational content into social solidarity and positioning participants as educators within the community [37]. In Nigeria, co-researchers who were living with skin-related NTDs demanded to be published by their names and pictures in order to claim the ownership of their stories [17]. Facilitators in Sabah employed iterative rounds and member-checking to ensure that participants were able to refine or challenge interpretations [25]. These processes reflect photovoice’s second aim, using dialogue to move beyond description toward collective meaning-making. When effectively facilitated, discussions allowed participants to connect personal experiences to broader structural conditions, including access to services, local governance, and gender norms.

However, critical dialogue was not automatic. Skilled facilitation is essential not only to keep conversations inclusive but to push participants beyond surface observations toward structural critique [38]. Many studies provided limited detail on facilitation strategies or how power dynamics within groups were addressed. In the absence of specific approaches to link dialogue to collective problem-solving, conversations risked remaining descriptive rather than transformative [39]. This highlights a key challenge of photovoice in moving discussions from the recording of consensus to critical consciousness, which is necessary to confront structural neglect effectively.

### Reaching policymakers for action and change

The third aim of photovoice is engagement with policymakers. Few studies went beyond community-level dialogue to influence policy. Exhibitions, community meetings, and photo booklets were the common outputs [17,19]. The ability to visually represent community structures and issues through participatory approaches such as photovoice can result in more effective implementation strategies [40–42]. The challenge is not in producing evidence but integrating it into decision-making spaces. In the Netherlands, children’s stories about poverty have reached policymakers but fall short of influencing action, pointing to a disparity between visibility and uptake [43]. Without advocacy planning, alignment with policy cycles, and institutional partnerships, photovoice risks being confined to awareness-raising. Translating photovoice evidence into actionable policy is possible when advocacy channels are built; in Madrid, photovoice informed policy recommendations on improving urban environments for physical activity [44].

### Strength and limitation

To our knowledge, this is the first synthesis of photovoice applications across NTD research and related environmental and vector-borne contexts, extending beyond single diseases to shared determinants affecting marginalised populations. By intentionally incorporating WASH behaviours and malaria that intersect within the same vulnerable population, we widen the lens to the broader determinants. Framing Wang and Burris’ three foundational aims of photovoice as an evaluative lens proved to be a replicable framework for assessing the translation of participatory intent into practice and determining persistent gaps. Taken together, our findings highlight that photovoice can meaningfully reframe neglect in NTD research, but its impact depends on careful design across participation, dialogue, and policy engagement. For practice, this calls for inclusive recruitment strategies and investment in skilled facilitation to ensure diverse voices are heard. For policy, early alignment with decision-making processes and sustained partnerships is essential to translate visibility into actionable change. For research, future studies should adopt longitudinal and iterative designs, expand coverage to diverse NTDs and settings, and clearly document participatory processes to strengthen evidence on lasting impact.

Nevertheless, this scoping review has several limitations and should be interpreted carefully. First, the evidence base was small, with only 13 included studies, limiting the extent to which the findings can be generalised across all NTDs, endemic settings, or participatory visual research contexts. Second, the geographical distribution of studies was uneven, with most studies conducted in African settings and fewer studies from Southeast Asia, Latin America, and other endemic regions. Third, the included studies varied in methodological quality and in the depth of community participation. In some studies, photovoice functioned mainly as participatory data collection technique. Whereas in others, it was embedded more fully within community interpretation, dissemination, advocacy, or engagement with decision-makers. Fourth, the disease and determinant focus was heterogeneous. Malaria-related studies were retained because of their relevance to shared environmental and vector-borne determinants, although malaria is not classified as an NTD. Finally, most studies did not report long-term health, behavioural, programme, or policy outcomes. As a result, we could not determine the sustainability of photovoice-informed changes or whether visual evidence translated into sustained programme or policy influence. Future studies would benefit from clearer documentation of participatory processes, longer follow-up, and application across a broader range of NTDs, environmental determinants, and endemic settings to assess the versatility of photovoice as a strategy for confronting neglect.

## Conclusion

Reframing neglect in the context of NTDs and their related environmental and vector-borne determinants through photovoice is possible. Functioning both as a research method and an intervention, it centres affected communities, makes hidden realities visible, challenges stigma, and generates locally grounded evidences that ultimately advances equity in NTD prevention, control, and policy decision-making.

### Declaration of generative AI and AI-assisted technologies in the writing process

During the preparation of this work, the author(s) used Grammarly to support language refinement, grammar checking, and improvement of clarity and conciseness. A generative AI tool was also used to assist with the visual design of Fig 2. The substantive content of the manuscript and Fig 2, including study counts, categories, thematic interpretation, and conclusion, was developed, reviewed, clarified, and finalised by the author(s). The author(s) take full responsibility for the accuracy, integrity, and final content of the submitted manuscript.

## Supporting information

SI Checklist PRISMA-ScR checklist. Preferred reporting items for systematic reviews and meta-analyses extension for scoping reviews checklist for this scoping review.
(DOCX)

SI Table Full database-specific search strategies. Database-specific search strategies used to identify studies on photovoice applications in neglected tropical diseases and related environmental or vector-borne determinants.
(DOCX)
