# Supplementary material for: Reframing neglect in neglected tropical diseases and related environmental determinants: A scoping review of photovoice studies
Source: PLoS Negl Trop Dis. 2026 Aug 21;20(8):e0014655. doi: 10.1371/journal.pntd.0014655 (PMC13537680; doi:10.1371/journal.pntd.0014655)
Supplement: Supplementary file 2 [file pntd.0014655.s002.docx]

**SI Table. Full database-specific search strategies**

| **No** | **Database** | **Keyword Used** |
| --- | --- | --- |
| 1 | WOS | TS=(("Neglected Tropical Disease*" OR NTD* OR "Chagas Disease" OR "Human African Trypanosomiasis" OR Leishmaniasis OR "Taenia solium cysticercosis" OR Taeniosis OR Dracunculiasis OR Echinococcus OR "Foodborne trematod*" OR "Lymphatic Filariasis" OR Onchocerciasis OR Schistosomiasis OR "Soil-Transmitted Helminth*" OR Ascariasis OR "Hookworm Disease*" OR Trichuriasis OR Strongyloidiasis OR "Buruli Ulcer" OR Leprosy OR Hansen OR Trachoma OR Yaws OR Bejel OR Pinta OR Dengue OR Chikungunya OR Rabies OR Mycetoma OR Chromoblastomycosis OR "Deep Mycosis" OR Scabies OR Myiasis OR "Snakebite Envenoming")  AND  (Photovoice OR "Participatory Photography" OR "Visual Participatory" OR "Community Photography" OR "Action Research with Photography" OR "Visual Advocacy")  AND  (Prevent* OR Control OR Change OR Empower* OR "Community Engagement" OR "Community Participation" OR "Community-Based Intervention*" OR Advocacy OR "Health Promotion")) |
| 2 | Scopus | TITLE-ABS-KEY (("Neglected Tropical Disease*" OR NTD* OR "Chagas Disease" OR "Human African Trypanosomiasis" OR Leishmaniasis OR "Taenia solium cysticercosis" OR Taeniosis OR Dracunculiasis OR Echinococcus OR "Foodborne trematod*" OR "Lymphatic Filariasis" OR Onchocerciasis OR Schistosomiasis OR "Soil-Transmitted Helminth*" OR Ascariasis OR "Hookworm Disease*" OR Trichuriasis OR Strongyloidiasis OR "Buruli Ulcer" OR Leprosy OR Hansen OR Trachoma OR Yaws OR Bejel OR Pinta OR Dengue OR Chikungunya OR Rabies OR Mycetoma OR Chromoblastomycosis OR "Deep Mycosis" OR Scabies OR Myiasis OR "Snakebite Envenoming")  AND  (Photovoice OR "Participatory Photography" OR "Visual Participatory" OR "Community Photography" OR "Action Research with Photography" OR "Visual Advocacy")  AND  (Prevent* OR Control OR Change OR Empower* OR "Community Engagement" OR "Community Participation" OR "Community-Based Intervention*" OR Advocacy OR "Health Promotion")) |
| 3 | PubMed | (("Neglected Tropical Disease" OR NTD OR "Chagas Disease" OR "Human African Trypanosomiasis" OR Leishmaniasis OR "Taenia solium cysticercosis" OR Taeniosis OR Dracunculiasis OR Echinococcus OR "Foodborne trematod*" OR "Lymphatic Filariasis" OR Onchocerciasis OR Schistosomiasis OR "Soil-Transmitted Helminth*" OR Ascariasis OR "Hookworm Disease*" OR Trichuriasis OR Strongyloidiasis OR "Buruli Ulcer" OR Leprosy OR Hansen OR Trachoma OR Yaws OR Bejel OR Pinta OR Dengue OR Chikungunya OR Rabies OR Mycetoma OR Chromoblastomycosis OR "Deep Mycosis" OR Scabies OR Myiasis OR "Snakebite Envenoming")**  AND  (Photovoice OR "Participatory Photography" OR "Visual Participatory" OR "Community Photography" OR "Action Research with Photography" OR "Visual Advocacy")  AND  (Prevent OR Control OR Change OR Empower OR "Community Engagement" OR "Community Participation" OR "Community-Based Intervention*" OR Advocacy OR "Health Promotion")) |
| 4 | Ovid SP | ("Neglected Tropical Disease*" OR NTD* OR "Chagas Disease" OR "Human African Trypanosomiasis" OR Leishmaniasis OR "Taenia solium cysticercosis" OR Taeniosis OR Dracunculiasis OR Echinococcus OR "Foodborne trematod*" OR "Lymphatic Filariasis" OR Onchocerciasis OR Schistosomiasis OR "Soil-Transmitted Helminth*" OR Ascariasis OR "Hookworm Disease*" OR Trichuriasis OR Strongyloidiasis OR "Buruli Ulcer" OR Leprosy OR Hansen OR Trachoma OR Yaws OR Bejel OR Pinta OR Dengue OR Chikungunya OR Rabies OR Mycetoma OR Chromoblastomycosis OR "Deep Mycosis" OR Scabies OR Myiasis OR "Snakebite Envenoming").tw.  AND  (Photovoice OR "Participatory Photography" OR "Visual Participatory" OR "Community Photography" OR "Action Research with Photography" OR "Visual Advocacy").tw.  AND  (Prevent* OR Control OR Change OR Empower* OR "Community Engagement" OR "Community Participation" OR "Community-Based Intervention*" OR Advocacy OR "Health Promotion").tw. |
| 5 | EBSCOhost | (TI "Neglected Tropical Disease*" OR AB "Neglected Tropical Disease*" OR SU "Neglected Tropical Disease*" OR  TI NTD* OR AB NTD* OR SU NTD* OR  TI "Chagas Disease" OR AB "Chagas Disease" OR SU "Chagas Disease" OR  TI "Human African Trypanosomiasis" OR AB "Human African Trypanosomiasis" OR SU "Human African Trypanosomiasis" OR  TI Leishmaniasis OR AB Leishmaniasis OR SU Leishmaniasis OR  TI "Taenia solium cysticercosis" OR AB "Taenia solium cysticercosis" OR SU "Taenia solium cysticercosis" OR  TI Taeniosis OR AB Taeniosis OR SU Taeniosis OR  TI Dracunculiasis OR AB Dracunculiasis OR SU Dracunculiasis OR  TI Echinococcus OR AB Echinococcus OR SU Echinococcus OR  TI "Foodborne trematod*" OR AB "Foodborne trematod*" OR SU "Foodborne trematod*" OR  TI "Lymphatic Filariasis" OR AB "Lymphatic Filariasis" OR SU "Lymphatic Filariasis" OR  TI Onchocerciasis OR AB Onchocerciasis OR SU Onchocerciasis OR  TI Schistosomiasis OR AB Schistosomiasis OR SU Schistosomiasis OR  TI "Soil-Transmitted Helminth*" OR AB "Soil-Transmitted Helminth*" OR SU "Soil-Transmitted Helminth*" OR  TI Ascariasis OR AB Ascariasis OR SU Ascariasis OR  TI "Hookworm Disease*" OR AB "Hookworm Disease*" OR SU "Hookworm Disease*" OR  TI Trichuriasis OR AB Trichuriasis OR SU Trichuriasis OR  TI Strongyloidiasis OR AB Strongyloidiasis OR SU Strongyloidiasis OR  TI "Buruli Ulcer" OR AB "Buruli Ulcer" OR SU "Buruli Ulcer" OR  TI Leprosy OR AB Leprosy OR SU Leprosy OR  TI Hansen OR AB Hansen OR SU Hansen OR  TI Trachoma OR AB Trachoma OR SU Trachoma OR  TI Yaws OR AB Yaws OR SU Yaws OR  TI Bejel OR AB Bejel OR SU Bejel OR  TI Pinta OR AB Pinta OR SU Pinta OR  TI Dengue OR AB Dengue OR SU Dengue OR  TI Chikungunya OR AB Chikungunya OR SU Chikungunya OR  TI Rabies OR AB Rabies OR SU Rabies OR  TI Mycetoma OR AB Mycetoma OR SU Mycetoma OR  TI Chromoblastomycosis OR AB Chromoblastomycosis OR SU Chromoblastomycosis OR  TI "Deep Mycosis" OR AB "Deep Mycosis" OR SU "Deep Mycosis" OR  TI Scabies OR AB Scabies OR SU Scabies OR  TI Myiasis OR AB Myiasis OR SU Myiasis OR  TI "Snakebite Envenoming" OR AB "Snakebite Envenoming" OR SU "Snakebite Envenoming")  AND  (TI Photovoice OR AB Photovoice OR SU Photovoice OR  TI "Participatory Photography" OR AB "Participatory Photography" OR SU "Participatory Photography" OR  TI "Visual Participatory" OR AB "Visual Participatory" OR SU "Visual Participatory" OR  TI "Community Photography" OR AB "Community Photography" OR SU "Community Photography" OR  TI "Action Research with Photography" OR AB "Action Research with Photography" OR SU "Action Research with Photography" OR  TI "Visual Advocacy" OR AB "Visual Advocacy" OR SU "Visual Advocacy")  AND  (TI Prevent* OR AB Prevent* OR SU Prevent* OR  TI Control OR AB Control OR SU Control OR  TI Change OR AB Change OR SU Change OR  TI Empower* OR AB Empower* OR SU Empower* OR  TI "Community Engagement" OR AB "Community Engagement" OR SU "Community Engagement" OR  TI "Community Participation" OR AB "Community Participation" OR SU "Community Participation" OR  TI "Community-Based Intervention*" OR AB "Community-Based Intervention*" OR SU "Community-Based Intervention*" OR  TI Advocacy OR AB Advocacy OR SU Advocacy OR  TI "Health Promotion" OR AB "Health Promotion" OR SU "Health Promotion") |
| 6 | Sage Journal | ("Neglected Tropical Disease*" OR NTD* OR "Chagas Disease" OR "Human African Trypanosomiasis" OR Leishmaniasis OR "Taenia solium cysticercosis" OR Taeniosis OR Dracunculiasis OR Echinococcus OR "Foodborne trematod*" OR "Lymphatic Filariasis" OR Onchocerciasis OR Schistosomiasis OR "Soil-Transmitted Helminth*" OR Ascariasis OR "Hookworm Disease*" OR Trichuriasis OR Strongyloidiasis OR "Buruli Ulcer" OR Leprosy OR Hansen OR Trachoma OR Yaws OR Bejel OR Pinta OR Dengue OR Chikungunya OR Rabies OR Mycetoma OR Chromoblastomycosis OR "Deep Mycosis" OR Scabies OR Myiasis OR "Snakebite Envenoming")  AND  (Photovoice OR "Participatory Photography" OR "Visual Participatory" OR "Community Photography" OR "Action Research with Photography" OR "Visual Advocacy")  AND  (Prevent* OR Control OR Change OR Empower* OR "Community Engagement" OR "Community Participation" OR "Community-Based Intervention*" OR Advocacy OR "Health Promotion") |
